# Splitting Atomic Minds: Hanna Segal and the Fear of Nuclear War in 1980S Britain

**DOI:** 10.3366/pah.2024.0493

**Published:** 2024-04-11

**Authors:** Hannah Proctor

**Affiliations:** Glasgow, UK

**Keywords:** psychoanalysis, Hanna Segal, Cold War, Greenham Common, nuclear weapons, British history, 1980s, Ronald Frasers

## Abstract

In 1985, British psychoanalyst Hanna Segal delivered the paper ‘Silence is the Real Crime’ to the first meeting of the group International Psychoanalysts Against Nuclear Weapons in Hamburg, appealing to her fellow analysts to counteract the denial of the geopolitical realities that characterized the late Cold War by intervening in public debates regarding the threat of nuclear war. A year later she gave a paper in London discussing clinical cases of patients who brought their nuclear anxieties to the couch. This article considers Segal’s political and clinical writings on the psychological consequences of the atomic age, situating them in the context in which she was living, writing and practising as an analyst: 1980s Britain in a moment of ‘nuclear anxiety’. I argue that Segal’s anti-nuclear writings shed light on what she called the ‘very very tricky’ relationship between psychoanalysis and politics. Segal confronted the tension between maintaining clinical neutrality in the consulting room while publicly expressing her political commitments, wrestling with the complex relationships between individuals and the societies in which they live.

The effects of nuclear weapons lie in our heads as well as in radioactive fallout. The damage that is being done *now* to people’s vision of the future and their faith in future generations is incalculable.

Alice Cook and Gwyn Kirk, *Greenham Women Everywhere*, 1983, p. 26

In famous lines from his 1965 preface to a new edition of *The Divided Self*, which had first been published in 1959, British anti-psychiatrist R.D. Laing describes a 17-year-old patient who told him ‘she was terrified because the Atom Bomb was inside her’.^1^ Laing then offers the following interpretation, insisting that this individual experiencing psychosis may be less mad than the nuclear society in which her delusion originated: ‘The statesmen of the world who boast and threaten that they have Doomsday weapons are far more dangerous, and far more estranged from “reality” than many of the people on whom the label “psychotic” is fixed’ (Laing, 1965, p. 12).

The notion that Cold War Britain overshadowed by the threat of nuclear war was a mad place that induced insanity in its citizens was not confined to the anti-psychiatry movement. The official magazine of the Campaign for Nuclear Disarmament (CND) was called *Sanity*, the antithesis of the acronym for the military doctrine of mutual assured destruction: MAD. Sentiments similar to Laing’s were also expressed by women involved in the peace movement:

We are facing the ultimate insanity – destroying the world we live in. This insanity is the ‘real’ world. Yet if women talk plainly about how this insanity affects their lives now, before the event, they are called mad themselves. (Cook & Kirk, 1983, p. 19)

The same text cites a letter from a woman who described working in a psychiatric hospital with a patient whose nuclear anxieties she presented not only as a symptom but as a cause of mental distress:

One woman I was working with was having nightmares every night about the nuclear disaster, the end of the world, and it meant that she just couldn’t carry on with her ordinary life because it was meaningless to her, because she felt so hopeless, that there was nothing she could do about it. She’d collapsed under that pressure and had been labelled mad and locked away because of it. Supposedly for her protection, she’d been pumped full of drugs and put away from society.

She *knew* the reality. She just let it in and that was why it was so devastating for her, because she was isolated in that position […] She wasn’t mad to be having breakdowns about it. We should all be changing society so no one needs to have a breakdown. (Cook and Kirk, 1983, pp. 19–20)

Laing was writing in the mid-1960s, in the period just after the Cuban missile crisis of 1962, whereas the above citation comes from Alice Cook and Gwyn Kirk’s *Greenham Women Everywhere: Dreams, Ideas and Actions from the Women*’*s Peace Movement*, first published in 1983, a moment of renewed nuclear anxiety and anti-nuclear activism in Britain.

In the context of the breakdown of the détente between the USA and USSR, at a meeting of NATO (North Atlantic Treaty Organization) ministers in Brussels on 12 December 1979 it was announced that US ground-launched nuclear cruise missiles would be placed in Western Europe, in what was known as the ‘NATO Double Track Decision’. Britain agreed to take 160 weapons. Margaret Thatcher had been elected Prime Minister seven months earlier on a manifesto pledging to ‘increase the importance of ensuring the continuing effectiveness of Britain’s nuclear deterrent’ (Conservative Party Manifesto, 1979). The period Daniel Cordle has called the ‘nuclear 1980s’ ran for slightly longer than the decade: from December 1979 through to the collapse of state socialism across the Eastern bloc between 1989 and 1991 (Cordle, 2017).

During this period, ‘nuclear anxiety’ pervaded British discourse: artistic, literary and pop cultural representations of post-nuclear scenarios proliferated and there was a resurgence of anti-nuclear activism (Hogg, 2016, p. 11).^2^ Following the screening of the docudrama *Threads* on BBC TV in September 1984, which imagined the impact of a nuclear war on Britain, many viewers reported suffering from insomnia and nightmares (Cordle, 2013). Membership of CND in Britain, which had dwindled in the 1970s, grew rapidly in the early 1980s and a range of other peace, anti-nuclear and environmental groups were founded. The title of social historian E.P. Thompson’s co-edited 1980 collection *Protest and Survive* was a twist on the infamously scaremongering government-issued pamphlet *Protect and Survive* produced between 1974 and 1980. ‘We must protest if we are to survive’, Thompson proclaimed urgently (Thompson, 1980, p. 30). Anti-nuclear rallies attracted tens of thousands of people, and peace camps, from Faslane in the west of Scotland to Greenham Common in Berkshire, were established across Britain (Fairhall, 2006).

## The Atomic Unconscious?

Though psychoanalytic theory was not central to nuclear discourses, psycho-analysts in Britain also addressed the pervasive fears of atomic warfare during this period of heightened nuclear anxieties and resurgent anti-nuclear activism. This article explores political and clinical writings by the Polish-born British psycho-analyst Hanna Segal, specifically focusing on two papers she first delivered in the mid-1980s, which confront anxieties induced by the existence of nuclear weapons and the possibility of nuclear warfare. It considers these publications alongside Cook and Kirk’s *Greenham Women Everywhere* and Ronald Fraser’s 1984 memoir *In Search of a Past*, all of which emerged from the particular social environment of 1980s Britain. Segal published papers that addressed the nuclear threat from a theoretical perspective but she also described seeing patients who spoke of their fears of nuclear war on the couch. She took a psychoanalytic approach to the themes explored by Cook and Kirk. Her work on nuclear nightmares, the feelings of mass helplessness brought on by the threat of nuclear war, and possible ways of overcoming such feelings by intervening in public debate to change government policy also reveal the challenges that attend attempts to apply psychoanalytic models to political issues more broadly.

Segal was almost seventy years old in 1987 when she published ‘Silence is the Real Crime’, her major paper on the role psychoanalysts might play in confronting the reality of the nuclear threat. She published various additional papers addressing geopolitical issues between then and her death in 2011. Though she described her anti-nuclear papers as her first publicly political interventions as a psychoanalyst, she had long been committed to social justice, which she attributed to her ‘identification with the underdog’ (Rose, 1990, p. 201). Born in Łódź, Poland in 1918 into a secular Jewish family, Segal first read Freud while attending international school in Geneva in adolescence, where her father worked as a journal editor for the League of Nations. She returned to Poland alone at the age of twelve, where she became ‘quite close to the Trotskyists’ as an activist involved in the Polish Socialist Party and the youth movement against the right-wing government (Quinodoz, 2007, p. 8). Forced to leave Switzerland due to the anti-fascist stance of the League of Nations’ journal, Segal’s parents fled to France. Segal was visiting her parents in Paris in 1939 when Hitler invaded Poland; she was unable to return. While in Paris she saw Picasso’s *Guernica* and recalled that three years earlier her parents had caught her attempting to sneak out of the house to join a friend fighting on the Republican side in the Spanish Civil War (Quinodoz, 2007, p. 10; Steiner, 2012, p. 459).

When the Nazi occupation of France began in 1940 she and her family fled to Britain, a decision she felt torn about at the time, as it meant that she had chosen not to join her ‘comrades in the Resistance’ (Quinodoz, 2007, p. 10). She later described herself in her youth as a ‘bit of a do-gooder’ who ‘wanted to be of social use in the world’ (Pick & Roper, 2000, p. 168). During her medical studies in Edinburgh in 1941, she encountered the analyst Ronald Fairbairn, through whom she first heard of Melanie Klein. She persuaded Klein to take her on as an analysand in London, completed her training analysis in 1946 and at twenty-nine became the youngest member of the British Psychoanalytical Society. Although she trained with Klein and continued to work in the Kleinian tradition, Segal maintained that she remained largely oblivious to the ‘Controversial Discussions’ raging between the rival factions associated with Anna Freud and Klein at the time she first encountered the British Psychoanalytical Society (Pick, 2001, p. 2). Her major publications over the ensuing decades included introductions to Klein’s work, engagements with dreams, literature and aesthetics, as well as writings on psychosis and the elderly based on her extensive clinical work (she continued seeing patients into her late eighties) (Henley, 2008).

Alongside increased political activism, the 1980s also saw a surge of global campaigning among medical professionals, including many based in Britain, who opposed nuclear warfare and sought to raise awareness of the deleterious health effects of radiation. Psychiatrists and psychologists were central participants in these initiatives, which considered the potential psychic toll that could result from nuclear war, as well as addressing the anxieties associated with living with its possibility (Chivian *et al*., 1982; Thompson, 1985; White, 1986). Unlike major medical organizations such as International Physicians for the Prevention of Nuclear War (IPPNW) and Physicians for Social Responsibility, who were the co-winners of the Nobel Peace Prize in 1985, Segal was co-founder (with Moses Laufer) of the far smaller and more marginal group International Psychoanalysts Against Nuclear Weapons, whose relatively negligible public influence reflected the dwindling dominance of psychoanalysis within the ‘psy’ disciplines and in popular culture during the late Cold War (Woodward, 1986).

Segal’s writings on nuclear war, unlike those of psychiatrists such as Robert Jay Lifton (whose writings on Hiroshima she engaged with), did not have significant policy implications or reach wide non-specialist contemporary audiences. Segal’s anti-nuclear writings remain significant nevertheless, this article will argue, not only as historical artefacts reflecting pervasive nuclear anxieties in 1980s Britain, but as attempts to apply psychoanalytic methods and concepts to geopolitical issues. Unlike much of the existing canonical psychoanalytic work on the psychological damage inflicted by the First and Second World Wars (and unlike Lifton’s publications on survivors of Hiroshima and Nagasaki), Segal’s writings on nuclear weapons in the late Cold War deal not with traumatic past experiences but with anxieties about potential devastation and destruction in the future.

## Group Psychosis

In ‘Silence is the Real Crime’, originally presented as a paper to the first meeting of the group International Psychoanalysts Against Nuclear Weapons in Hamburg in 1985, and subsequently published in the *International Review of Psychoanalysis* in 1987, Hanna Segal attempts to unpick the denial that prevents people from confronting the reality of the consequences of nuclear war. She describes the resistance to knowledge, or ‘disavowal’, in which people simultaneously know and deny the possibility of nuclear war, refusing to visualize the end of the world while glibly discussing nuclear weapons in euphemistic language. This, she claims, is an example of splitting, a concept introduced by Freud to describe a process in which, as Segal explains elsewhere, the ‘ego splits itself so that there is one part, the normal ego, which takes account of reality, and another which, under the influence of the instincts, detaches itself from reality’ (Segal, 1981, p. 15). People accept that nuclear war is possible, even likely, and know that if it happens few will survive: ‘And yet the same vast majority live their lives in that shadow without taking active steps to change policy’ (Segal, 1997[1987], p. 119).

In the paper, Segal deploys a rhetoric that plays with the boundaries between metaphorical and literal ‘madness’ and social and individual ‘madness’. As she describes, ‘we observe a phenomenon that is more like a surrealist scenario, an unbearable nightmare or a psychosis than a sane world’ (Segal, 1997[1987], p. 117). Her paper is addressed to her fellow psychoanalysts, whom she implores to take ‘active efforts to halt what I consider to be a mad process’ (p. 117), arguing that psychoanalysts are well placed to offer insights into the psychic phenomena that are of relevance to a nuclear age, such as aggression, self-destruction and denial.

Her subject necessitates moving between distinct scales, and shifting from discussions of individuals to discussions of groups. In order to make sense of the dehumanization of the enemy on a national level, for example, she invokes the idea of regression to a paranoid-schizoid position on the individual level. In Segal’s gloss of Melanie Klein, the depressive position is ‘characterized by a capacity to recognize one’s own aggression, and to experience guilt and mourning’, whereas the paranoid-schizoid position is ‘characterized by the operation of denial, splitting and projection’ (Segal, 1997[1987], p. 120). Yet she concedes that such an observation cannot simply be transferred to groups; the observation is an analogy rather than a diagnosis. Indeed, she recognizes that groups might act in ‘mad’ ways despite being comprised of ‘sane’ individuals, giving the example of ‘born-again’ Christians in the United States being convinced that a God-willed nuclear war would usher in a new world order. Drawing on Wilfred Bion’s distinction between the ‘work group’, which is oriented towards reality, and the ‘basic assumption’ group, which tends towards psychosis, she argues that the latter, which emerges in ‘situations of excessive anxiety’, prevails in national attitudes to nuclear war (p. 121).

Segal claims that the potential annihilation represented by the existence of nuclear weapons is qualitatively distinct from the threats posed by other kinds of warfare. The almost unreal capacity for destruction in an atomic age increases, as she puts it, the ‘lure of omnipotence’ (Segal, 1997[1987], p. 123). The desire for Armageddon among born-again Christians, and their association of total destruction with divine salvation, reveals, for Segal, the operation of the death instinct, which she understands, following Freud, as a desire to return to an inorganic state. But, beyond this, she suggests that the extreme views of such groups reveal something about broader cultural attitudes to nuclear war. The example of born-again Christianity functions, for Segal, like a lens that magnifies the rule, rather than being the exception that proves it.

For Segal, living in a world with atomic weapons ‘mobilizes and actualizes the world of the schizophrenic’ because it destroys the ‘boundaries between reality and phantasy’ (Segal, 1997[1987], p. 123). By this she seems to mean that something almost unfathomable or fantastical has become part of reality as a result of the existence of these weapons, but also that the destructive realities of the nuclear world activate unconscious ‘wishes of annihilation’ (p. 123). Some of Segal’s earliest publications drew on her clinical work with psychotic patients, which she completed while working alongside Herbert Rosenfeld, another of Klein’s students, and Bion who was also training with Klein in the late 1940s (Bell, 2015). ‘Some Aspects of the Analysis of a Schizophrenic’, which described the innovative approach to transference she developed with her patient ‘Edward’, was presented to the British Psychoanalytical Society in 1949 as her membership paper, and published in 1950. Edward had recently returned from India, where he had been sent to train as an engineer with the Officer Cadet Training Unit (OCTU) during the Second World War. He suffered a breakdown whilst working in a darkroom after failing to get commissioned by the OCTU, and spent six months in military hospitals experiencing ‘delusions, nightmares, hallucinations’ (Segal, 1950, p. 268). Segal describes Edward’s experience of psychosis, which was prompted by his experiences during the war, as being characterized by a fear of destruction combined with an incapacity to distinguish between himself and the external world: ‘his own destruction being equivalent to the destruction of the whole world’ (p. 269), a description echoed in her discussions of the fear of nuclear war almost four decades later.

In ‘Silence is the Real Crime’, Segal takes the extreme beliefs of born-again Christians to reveal something more general about social attitudes to the nuclear threat, and she similarly suggests that her clinical work with psychotics can provide insights into the terrors provoked by the prospect of nuclear war across society: ‘In the depths of our unconscious […] such unintegrated wishes and terrors still exist. We are all only partly sane, and such circumstances as prevail now mobilize the most primitive parts of ourselves’ (Segal, 1997[1987], p. 123). The challenge, then, is how the life instinct might succeed in taming the forces of destruction in the context of a potentially deadly world.

Segal offers two distinct, yet interrelated, answers to this question: one clinical, the other political. As a psychoanalyst in the consulting room, Segal is able to help her patients deal with their desire to annihilate or to be annihilated. She also appeals to analysts to acknowledge their own destructive and self-destructive tendencies, but she recognizes that therapeutic work focused on overcoming denial on an individual level is insufficient for addressing the nuclear threat. ‘Silence is the Real Crime’ is also motivated by her desire to effect political change by making the case for nuclear disarmament, and contributing to a social reckoning with a violent external reality. The death drive is central to both Segal’s political and clinical writings on nuclear war, and her discussions of the relationship between the life and death instincts are key to her vision for overcoming both individual and social hopelessness: ‘We must try to find means to mobilize our life forces against the destructive powers’ (Segal, 1997[1987], p. 127). But her clinical goal is distinct from her political one: the former involves coming to terms with external reality, while the latter aims to change it.

## Annihilation and Survival

Nuclear fission was discovered in December 1938 – a few months after Freud fled Vienna for London, and a few months before he died, which was six years before atomic bombs were detonated over Hiroshima and Nagasaki. Nuclear fission is named analogically. Its name is a biological analogy that compares the process to that of living cells. In *Beyond the Pleasure Principle* (1920) Freud describes a longing to re-establish an original unity, whereas nuclear fission splits an atom into two or more smaller nuclei, in a process that releases energy.

In *Beyond the Pleasure Principle*, death is located not only in the future but also in the past. It is not just an individual event, but a prior state that preceded the existence of individual organisms; death preceded life as such. *Beyond the Pleasure Principle* is more concerned with an eternity of darkness in the past, whilst Segal’s writings on the prospect of nuclear war focus on the future. For Freud, inorganic nature preceded life, and living organisms strive to return to their deathly origins. The implicit existence of a lifeless pre-prelapsarian universe is therefore key to the strange narrative Freud presents:

The attributes of life were at some time evoked in inanimate matter by the action of a force of whose nature we can form no conception […] The tension which then arose in what had hitherto been an inanimate substance endeavoured to cancel itself out. In this way the first instinct came into being: the instinct to return to the inanimate state. (Freud, 1920, p. 38)

Freud pictures a single cell adrift in a threatening inorganic environment, ‘a little fragment of living substance […] suspended in the middle of an external world charged with the most powerful energies’ (p. 27). To protect itself from the surrounding world, it forms ‘a protective shield against stimuli’ (p. 27). Wishing to die only its proper, ‘natural’ death, the fragile organism goes to great lengths to avoid perishing prematurely, and coats itself in an inorganic ‘crust’, a carapace of death. As more complex life forms evolved, this surface ‘crust’ was internalized, but its primal deathliness remained. The emergence of life in Freud’s peculiar account represents a violent break with an original inorganic unity. Eros, or the life instinct, strives to reinstate this lost wholeness, pulling back together that which has been torn asunder.

The discussions of the origins of life in *Beyond the Pleasure Principle* offer a kind of mirror image – though perhaps a more accurate metaphor would be that of a video tape played backwards – of the nuclear war scenario that preoccupied Segal. Where Freud looked to the beginning of life, Segal contemplates its possible end. Like Freud, she is thinking about life and death not only in individualized terms but in a totalizing sense. She sees the nuclear threat as representing the end of life on Earth, after which there would be ‘no meaningful survival’ (Segal, 1987] 1985], p. 117). The slightly back-to-front logic evident in Freud’s discussion of the organism wanting to die its own death – the strange argument that the death drive functions to preserves life – is also apparent in Segal’s discussion of nuclear deterrents, which she argues encourage destruction despite ostensibly existing to preclude it.

Segal was a Kleinian, and Klein’s understanding of the death drive differed from Freud’s. In Klein’s conceptualizations of the death drive, she imagined it in psychological rather than biological terms, transposing Freud’s discussion of simple organisms into a human register. That is, she addressed the origins of individual human lives and individual egos, rather than imagining the beginning of life as such. She was interested in the birth of human babies, rather than with the ‘coming to life of inorganic substance’ (Freud, 1920, p. 61). As Segal outlined in a book introducing the work of her former analyst, for Klein the death drive’s manifestations relate not to the organism, but to the ego: Klein ‘does not speak of an organism deflecting, but of a primitive ego projecting the death instinct’ (Segal, 1981, p. 116). In her 1957 essay ‘A Study of Envy and Gratitude’, Klein explicitly differentiates her understanding of the death drive from Freud’s in the following terms:

The threat of annihilation by the death instinct within is, in my view – which differs from Freud’s on this point – the primordial anxiety and it is the ego itself which, in the services of the life instinct, deflects to some extent that threat outwards. The fundamental defence against the death instinct Freud attributed to the organism, whereas I regard this process as the primary activity of the ego. (Klein, 1970, p. 216)

Pictured spatially, Freud’s image of the organism with its death-like inorganic ‘crust’, formed to protect itself from the onslaughts of the external world, is almost the inverse of this description, which sees the most terrifying and dangerous threats as coming from *within*, a process that starts at the very beginning of an individual’s life, preceding its interactions with external reality. Where Freud imagines a protective shield against attacks, Klein seems to suggest that the process of defence can function more like a weapon.

Klein had an expansive understanding of the ego, which she claimed could feel anxiety, and would attempt to deflect it from the moment of birth. Unlike Freud, Klein explicitly associated the death drive with anxiety, as Segal explains:

thinking in terms of a primitive ego, [Klein] maintains that the operation of the death instinct gives rise to a fear of annihilation, and that it is this basic fear which leads to the defensive projection of the death instinct […] The terror of disintegration and total annihilation is the deepest fear stirred by the operation of the death instinct within. (Segal, 1981, p. 116)

Against those who characterize Klein’s work as focusing on the internal at the expense of the external, Segal insists that Klein ‘constantly speaks of the environment’, such as in the famous case history of ‘Richard’, which discusses the emotional impact of an air raid on the little boy (Pick, 2006, p. 11). For Segal, the death drive might operate within, but it does not exist in isolation from the external environment. In ‘Silence is the Real Crime’, Segal equates the possibility of total destruction in nuclear war to the wish for annihilation that Klein associates with the death drive:

We can, at the push of a button, annihilate the world. In this world of primitive omnipotence, the problem is not of death wishes and a fear of death which pertain to the depressive and Oedipal world, it is governed by wishes for annihilation of, the self and the world. (1997[1987], p. 123)

Atomic annihilation is not like natural death – it is certainly not ‘dying one’s own death’ – it is not even like conventional warfare, because it ‘destroys the possibility of symbolic survival’: ‘The prospect of death in atomic warfare leaves an unimaginable void and produces terror of a different kind’ (p. 123).

This terror is strong, and it comes from an external threat, but Segal’s concern is not with imagining a fragile human being experiencing a nuclear blast. Indeed, she perceives that a ‘protective shield against stimuli’ would do nothing in the face of a nuclear war (to repeat Freud’s phrase). Nor, in spite of criticizing other people’s failure to visualize future possibilities, does Segal describe what a world in the wake of a nuclear blast might look like. Instead, she addresses how people protect themselves *psychically* from the knowledge of this possible threat without disavowing the threat altogether. In this case, the kind of ‘preparedness for anxiety’ Freud discusses would be possible (Freud, 1920, p. 31). Ultimately, she is more concerned with how people construct internal defences against knowledge, rather than external defences against reality. But it is still possible to discern a social implication to her arguments, because she suggests that a psychological confrontation with the real devastation nuclear weapons pose could ultimately lead to a transformation in political policy. As in her own defences of Klein, for Segal the subject’s internal reality cannot exist in isolation from their social environment, an external context that people are both shaped by and can act to shape. The question of *how* a transformation of the external world might be brought about does, however, remain obscure.

## Bombs on the Couch

Freud ventured ‘beyond the pleasure principle’ after hearing patients who had fought in the First World War recount traumatic dreams, and many theorists were drawn to the concept of the death drive in the aftermath of the Second World War, in attempts to contend with its traumas (Herzog, 2016). On a couch in late Cold War London, however, Segal’s patients reported fears of a possible future nuclear war. In her case histories, the threatened violence of the external world came to form part of the patient’s internal world. Freud addressed concrete past experiences, whereas Segal describes abstract terrors about the future in which the significance of nuclear war seems to have less to do with an actual threat of destruction located in the external world than it does with the patients’ internal destructive processes.

In ‘On the Clinical Usefulness of the Concept of the Death Instinct’, first delivered as a paper in London in 1986, Segal claims that since Freud first introduced the death drive in 1920, subsequent clinical developments have made it possible for psychoanalysts to clearly identify manifestations of the death drive in their patients ‘in an almost pure form in conflict with the life forces’ (Segal, 1997 [1986], p. 15). She not only identifies examples of the operation of the death drive, but maintains that identifying it in analysis can act as a turning point for the patient: ‘Beyond the pleasure principle, beyond ambivalence, aggression, persecution, jealousy, envy and so on, there is a constant pull of the self-destructive forces, and it is the task of the analyst to deal with them’ (p. 20). By going beyond the pleasure principle in the consulting room, the analyst can help encourage the life instinct to gain the upper hand.

Segal’s analysand, Mrs A., is of East European and West European descent, with a parent from each side of the Iron Curtain. She is obsessed with the possibility that the Cold War could turn hot. The implication is that the geopolitical divides structuring the world had a counterpart in her patient’s psyche, recalling D.W. Winnicott’s strange metaphor, which recurred in papers he wrote in the 1960s, that likened the individual psyche to Berlin divided by the Wall (Winnicott, 1990). In one session, Mrs A. describes her failure to attend a meeting of the Campaign for Nuclear Disarmament and expresses frustration with her own passivity. She then moves on to discuss her fear of nuclear war, before returning once more to the unattended meeting, claiming that she stopped herself from going because she felt anxious about being judged for attending by a friend. Segal remarks that during this part of the conversation, she ‘felt as though the analytic space was getting filled with persecutory objects of a very fragmentary kind’ (Segal, 1986, p. 16). When Segal asks her patient how she is feeling about an impending break in their sessions, Mrs A. returns to the image of the onset of nuclear war, snapping back: ‘I hate last sessions – I can’t stand them. I wish I could just push a button and make it disappear’ (p. 16).

Asked why she used this turn of phrase, why she wants to push the (nuclear) button, Mrs A. shifts her focus from the immediate dynamics of the analytic space to a possible future world beyond, revealing that she finds the idea of dying in a nuclear war far less terrifying than the idea of surviving one. Indeed, she claims that she positively wishes a nuclear war would happen, as long as she and her child were guaranteed to die immediately. The idea of surviving the end of the world as she knows it, of living in the ruins of what had been, of living amid the radioactive fallout, preoccupies the patient and fills her with fear. Segal describes her as ‘living in a mental post-war situation and subjected to a perpetual fall-out’ (Segal, 1986, p. 16). According to Segal, Mrs A.’s obsession with the possibility of nuclear war revealed ‘how she dealt with her death drive’:

Pushing the button was an expression of the death instinct, but combined with immediate projection, so that the threat of death was felt to come from outside – the fall-out. I think that in that session she got in touch with an almost direct experience of her own wish for total annihilation of the world and herself. (p. 16)

Segal claims that, after being offered this interpretation, Mrs A.’s sense of persecution diminished, and that she experienced a lasting sense of relief following this session. Mrs A. became less anxious and less destructive as a result of this confrontation with her own desire for total annihilation. This encounter with what Segal claimed was a manifestation of the death drive enabled the patient to continue living; indeed, it made the experience of living easier.

In an interview in 2000, Segal rejected the notion that dream symbols are universal: for her, ‘[s]ymbols carry a history with them’. Symbols change, she explains, because the social environments in which dreamers live also change. ‘Situations change, anxieties change. Takes dreams, let’s say, in adolescents confronted with endless unemployment or confronted with a nuclear threat’ (Pick & Roper, 2000, p. 166). In the case of Mrs A., however, the threat of nuclear war is treated as little more than a metaphor. The patient’s experience of living in the ‘nuclear 1980s’ appears only in the manifest content of the dream, which masks a latent anxiety about something else. Segal does not present her patient’s relationship to the prospect of annihilation as a social reality over which she had little control, but focuses on her patient’s own destructive impulses.

Later in the same essay, Segal turns to the case of another patient, Mr D., who describes a dream where the threat of nuclear war reappears:

There was an area in which everything and everybody was immobile and nearly dead. Around that area, at regular intervals, there were nuclear weapons facing outwards. If anybody approached the area the weapons would automatically trigger off. Amongst the near-dead people in the area were his parents. (Segal, 1986, p. 19)

Unlike Mrs A., who initially preferred death to survival, this dream is not about complete death but about near-death, not about annihilation but survival: ‘life is allowed to continue just so long as nothing is really alive and functioning’ (Segal, 1986, p. 19). As in the case of Mrs A,. Segal describes how identifying manifestations of the death drive in the consulting room could help the patient to confront his own problems with envy. In these clinical examples Segal associates the death drive with a desire for self-annihilation and the destruction of others, the appearance of which within the therapeutic setting can ultimately promote self-preservation. In neither case, however, does she ask how to move from the individual to the social, nor does she probe the significance of the political content of her patients’ revelations. Her patients may have felt some degree of relief about their personal situations following the sessions, but the actual threat of nuclear war was not diminished by doing so. Indeed, the actual threat of nuclear war does not figure in Segal’s interpretations of these two patients at all. There is no suggestion that Mrs A.’s ‘lasting sense of relief’ enabled her to become more actively involved in CND, for example. How can these clinical examples be connected to Segal’s insistence, in ‘Silence is the Real Crime’, that analysts should ‘contribute something to the overcoming of apathy’ regarding nuclear proliferation within society (Segal, 1997[1987], p. 127)?

## In Search of a Form

Segal wants to stop nuclear war from happening, and that means intervening in the social and political organization of the world. She explicitly states that clinical neutrality should not extend beyond the consulting room, arguing that it can function as a ‘shield of denial’, and encouraging psychoanalysts to take public political stands (Segal, 1997[1987], p. 127). Yet her argument in ‘Silence is the Real Crime’ is hard to reconcile with the clinical examples in ‘On the Clinical Usefulness of the Concept of the Death Instinct’. How can the grandiose political goal of abolishing nuclear weapons be combined with Segal’s small-scale role within the consulting room, in which patients must learn to keep living in a world where the nuclear threat remains? How to move from the clinical to the political? How to mobilize the life instinct against destruction on a social and not only on an individual level?

Segal acknowledges that the omnipotence represented by the existence of nuclear warheads goes hand in hand with a sense of helplessness and apathy for most people: ‘Confronted with the terror of the powers of destructiveness, we divest ourselves from our responsibilities by denial, projection and fragmentation’ (Segal, 1997[1987], p. 126). She sees the psychic responses to the threat of nuclear war as evidence of the operation of the death drive, but her clinical experiences in which patients were able to confront their destructive tendencies suggest that it is possible to at least partially overcome the desire to annihilate the world. Ultimately, her therapeutic work insists that integration – and hence a kind of fragile peace – is possible. Life can keep death in check. But although she argues that she wants people to acknowledge the reality – the external reality – of the bomb, as a psychoanalyst she can only help to defuse *internal* weapons.

The tension between managing terror on an individual level and fighting for peace on a political level recalls an insight from Dominick LaCapra’s contemporaneous essay ‘Psychoanalysis and History’ (1987), in which he observes that setting up an analogical relationship between the ontogenetic and the phylogenetic can close off the possibility of analysing the relationship between individual people and historical forces:

it is inadequate to rest content (as Freud sometimes did) with the analogy […] between the individual and society. Instead one must actively recognize that the analogy itself conceals the more basic interaction of psychoanalytic and sociocultural processes involving social individuals. (LaCapra, 1987, p. 242)

Segal does not set up this analogy explicitly, but it is evident in the tension between the two texts I have discussed here that political issues cannot be solved by clinical means. Segal’s therapeutic optimism is no cure for her social pessimism. Indeed, elsewhere she admits that even clinical hope can be accompanied by hopelessness precisely because it represents a moment in which patients ‘have to face reality’ (Segal, 1997[1987], p. 135).

Segal’s patient Mrs A. talked in her session about failing to attend a CND meeting for fear she would be judged by a friend. British social historian Ronald Fraser’s *In Search of a Past* (1984) includes an example of a similar discussion narrated from the other side of the analytic dyad. *In Search of a Past* is a memoir that switches between a narrative of Fraser’s personal experience of analysis, and material he recorded by conducting oral history interviews with former servants who worked for his parents at the stately home in which he grew up. Fraser attempts to understand himself as an individual formed by a particular social milieu at a particular moment in history, from the perspective of his two distinct roles: as analysand and as social historian. At one point during a session, Fraser describes telling his analyst that he had recently joined a demonstration against the nuclear arms race that went from the Greenham Common Women’s Peace camp to a weapons factory located near his childhood home.^3^ After this revelation, he recalls lying on the couch in silence wondering if his analyst is sympathetic to CND, and thinks: ‘Probably not. He’ll think it’s some kind of infantile defiance. Against every prescription, I refuse to say what I’m thinking’ (Fraser, 1984, p. 115). In another session, he clashes with his analyst about how he perceived his class position as a child: ‘I take comfort in the criticism that psychoanalysis refuses social reality –except to make you accept it’ (p. 112). He directly confronts his analyst about his suspicion that psychoanalysis is unable to account for the role material conditions play in forming subjectivity, proclaiming: ‘Your refusal of the social is a failure to recognize an important determinant’ (p. 112).

Segal could not be accused of failing to recognize the social in this manner. In an interview with Jacqueline Rose from 1990, Segal discussed the ‘very very tricky’ relationship between psychoanalysis and politics (Rose, 1990, p. 202). This trickiness, for Segal, primarily resides in the role of the analyst who should not impose their political convictions on their patients. She describes being concerned that making public statements on nuclear issues might interfere with her therapeutic relationships, but ultimately decided that the issue was too important to ignore. As well as stating that she would refuse to treat a fascist patient, and bemoaning the failures of psychoanalytic institutions to confront the rise of Nazism, she discusses the situation faced by psychoanalysts in Argentina during the military dictatorship as a more recent historical example in which an acknowledgement of political circumstances came into conflict with the psychoanalytic injunction to maintain clinical neutrality. The Argentinian Psychoanalytic Association (APA) remained officially politically neutral after the 1966 coup that established a dictatorship by a military junta. Many analysts refused to look beyond the consulting room despite the violent repressions and disappearances taking place beyond it; events that were impacting the lives of analysts and analysands alike (Hollander, 1990; Plotkin, 2001). This disavowal of the violent political situation led the analyst Marie Langer and others to take a stand, refusing to overlook the psychological consequences of the violent society in which they were practising. They split from the APA in 1971 to inaugurate a more socially engaged clinical practice, a decision Segal condones: ‘I do not believe that you could analyse someone well if thousands of people are disappearing and being tortured and it doesn’t play a part in the material’ (Rose, 1990, p. 202). But for all that she acknowledges that political realities can intrude upon the clinical encounter and demand that the analyst take a non-neutral attitude, Segal’s clinical writings discussing nuclear anxieties do not reflect explicitly on the psychic implications of the kinds of issues she discusses with Rose, or addresses in ‘Silence is the Real Crime’.

For Fraser, the CND demonstration is brought up not in relation to a discussion of the threat of nuclear war specifically, but in order to express his suspicions about the politically disengaged nature of psychoanalysis more generally. Fraser suspects his psychoanalyst will reduce his political commitment, his opposition to the threat posed by nuclear war, to an individual neurosis, pathologizing his decision to attend a demonstration. This, in turn, is connected to the relationship between himself as an individual and the social history he has lived through, which he is attempting to figure out through writing the book. That the book’s form is unable to smoothly accommodate both registers without switching between genres testifies to the intractability of the problem articulated by LaCapra. Segal’s ‘Silence is the Real Crime’ and ‘On the Clinical Usefulness of the Concept of the Death Instinct’ similarly operate in two distinct registers, but remain separate, the former addressed to external political conditions, the latter discussing inner psychic conflicts. *In Search of a Past*, by contrast, adopts a hybrid genre in its attempt to address the question of social and individual transformation simultaneously.

Sometimes Fraser and his analyst discuss the difficulties he has encountered in attempting to write about his own life as unfolding in history with a capital ‘H’. It takes Fraser time to realize that the two different pasts for which he is searching – the personal and the political – rely on one another, but cannot be neatly integrated into a single narrative. He struggles to bring the individual and the social together, but the form of the book reflects this difficulty rather than attempting to resolve it. As he remarks in the consulting room:

At first, I thought I wanted your help to overcome the difficulty of writing about the past; now I see that the difficulty is part and parcel of the past. This ‘voyage of inner discovery’, as I think you once called it, has to be combined with the account of the other voyage into the social past… And the two don’t always coincide… That’s my split vision. Formed by the past, a person is also deformed by it. (Fraser, 1984, p. 118)

He recognizes that psychoanalysis does not allow for a neat distinction to made between the internal and external, the psychic and social, or individuals and history, but remains exasperated by the lack of synthesis. By acknowledging that there is no easy analogy between the social and the individual, Fraser does not resolve the tension, but does at least describe it. Indeed, this irresolvable tension is the subject of his book. But finding a way to integrate the clinical with the political on the page – articulating that the social is not reducible to the individual, and that the internal is not a mere reflection of the external – leaves the question of political transformation unsolved. Fraser was attempting to make sense of the past, whereas Segal was seeking to intervene in the present for the sake of the future.

## From Individual Dreams to Collective Actions

The letters page of the autumn 1981 issue of *Sanity: The Magazine of CND* included the following message under the headline ‘FEARS’:

Dear Friends,

The worldwide escalation in arms expenditure and talk of ‘limited nuclear exchanges’ leads more and more people to active involvement in Peace movements, but how does that awareness affect other parts of our lives?

I am interested in the place that fear of nuclear war occupies in people’s imaginings.

I would like to hear from those who could contribute to this, particularly people who have dreamt about nuclear war. (Cook, 1981, p. 2)

The letter’s author, Alice Cook, who also claimed to have placed a similar appeal for dreams in the feminist magazine *Spare Rib* in 1980, later co-authored the book, already mentioned above, *Greenham Women Everywhere: Dreams, Ideas and Actions from the Women*’*s Peace Movement* (1983) with Gwyn Kirk.^4^ Written by two feminist activists in the peace movement less than two years after the Greenham Common women’s peace camp was established in September 1981, the book reflects on recent protests against cruise missiles, and non-violent tactics and direct action strategies adopted by women at Greenham and elsewhere in Britain. The camp banned men and sought to work non-hierarchically, in the hope of constructing an ‘alternative reality’ antithetical to the societies that produced nuclear weapons (Harford & Hopkins, 1984, p. 5).

The book’s second section. ‘A Private Nightmare’, quotes at length from letters describing dreams of nuclear war that were sent in response to Cook’s appeals. She claims that she felt compelled to gather such accounts from others because she was beset by nightmares about nuclear war and found it difficult to talk openly about her fears. She immediately received letters containing dreams, particularly from women, which she took as a testament to a pervasive social anxiety characterized by an inability to think about the future. Unlike in Segal’s clinical examples, where Mr D.’s dream of nuclear disaster is interpreted in relation to his own desire for self-annihilation, these dreams are read as direct manifestations of the kind of pervasive social terror Segal describes in ‘Silence is the Real Crime’. Cook and Kirk assume that the manifest content of the dreams is evidence of latent anxieties: ‘In dreams we may deal with information and feelings that we cannot assimilate in waking life’ (Cook & Kirk, 1983, p. 16).

Many of the dreams collated in the book unfold in the aftermath of an atomic blast in which the dreamer is one of few survivors in a destroyed radioactive landscape, surrounded by dead or damaged bodies. Others take place in the moments just before the bomb drops, when there is nothing that can be done by the dreamer to stop it. They often describe women searching helplessly for their husbands, children or friends. In addition to describing scenes, landscapes and events, many of the dreamers also dwell on their emotions, either their feelings in the dream itself or upon waking. Overwhelmingly, the letters speak of helplessness and isolation, ‘desperation and pessimism’ (Cook & Kirk, 1983, p. 13). Some speak of fear, some of numbness, others of a contradictory mixture of emotions, including acceptance and occasionally even thankfulness for the existence of a world prior to destruction:

It makes me feel empty and desolate, like stone. (p. 21)

I remember vividly the feeling […] total horror and panic, and yet tolerance at the same time. (p. 19)

I was startled to find that my predominant emotion was that of wonder and gratitude that I had been able to see those beautiful leaves before the world died. (p. 16)

Cook and Kirk identify the experience of being both emotionally frozen and frozen to the spot as a common trope in the dreams, which they interpret as a desire to block out the reality of the nuclear threat.

The authors, as well as some of the letter writers they cite, discuss the problem of denial in terms that echo Segal’s essay. A woman called Jayne accuses politicians of actively participating in hiding the deathly reality of the situation from the population, while also suggesting that politicians are personally unable to confront the reality themselves. She uses the term ‘splitting off’ to refer to the process people adopt to live with the fear of nuclear weapons but claims this compartmentalization produces neuroses that will worsen if the reality is not confronted (Cook & Kirk, 1983, p. 20). Like Segal, the authors also emphasize the role played by euphemistic or abstract language in creating an atmosphere of denial, rendering mass death unreal.

Though *Greenham Women Everywhere* does not mention Freud or psycho-analysis by name, Cook and Kirk invoke the psychoanalytic concept of ‘reality testing’ in a passage unpicking the significance of dreams about nuclear war:

One of the ways we normally differentiate ourselves from our dreams is by a process of reality testing: we test the reality of the world we have woken up into against the reality of the dream world we have left. We wake up and realise that the monsters who pursued us such a short time ago are not actually in the room. We perceive that our life is not actually threatened in the way it seemed in the dream. (Cook & Kirk, 1983, p. 21)^5^

Dreams about nuclear war are distinct from dreams about monsters, they argue, because ‘there is no waking up from this nightmare’; the nuclear threat is real. This is a similar observation to Segal’s comparison of the current social situation to madness; psychosis is a failure of reality testing (Rycroft, 1972, p. 153). In one of the letters sent to Cook, a woman called Carol describes experiencing a breakdown in which she succumbed to the anxiety she associates with her dreams of nuclear war. She describes herself as facing an impossible choice between a subjective madness that remains in touch with the realities of the world or a superficial sanity in which the nuclear threat is accepted or ignored. During her breakdown, she claims that she ‘wanted to be a victim of fear and panic, that it was somehow easier than the other choice’ (Cook & Kirk, 1983, p. 19). But the book’s authors go on to propose an alternative to these two equally impossible-seeming options.

A woman identified in the book as Ruth describes herself in a dream mourning for the dead, while also anticipating future deaths, including her own:

All I want is to find someone and die with someone I know. I always feel an incredible desolation, a sense of loss, that all these people are going to be dead in a short while. (Cook & Kirk, 1983, p. 13)

Cook and Kirk observe that, like Ruth, who dreams of seeking a companion with whom to die, many of the letters’ authors express feelings of loneliness and isolation. But they also frame the emergence and growth of the women’s peace movement shortly afterwards as an antidote to the atomization of the atomic age. They argue that since receiving the dream-filled letters many women had swapped individualized worrying for collective action in the peace movement, enabling them to relate differently to their fears: ‘We have to try to come to terms with these dreadful feelings of despair and paralysis or else we are submerged by them’ (Cook & Kirk, 1983, p. 14). Accounts of nightmares also feature in secondary accounts of the camp. David Fairhall cites an activist who was spurred to action after being haunted by dreams of waking up to a view of a destroyed London, while Sasha Roseneil quotes a Greenham woman whose inability to sleep drove her into activism, which ultimately soothed her fears:

I got involved basically because I was frightened to go to sleep. I kept having nightmares about nuclear war. And it was so real. I’d feel the heat on my back in the nightmare, and the skin on my back coming off in my hands. It was that real that I was afraid to go to sleep. So I joined CND, and it didn’t go away. I did Porth Square [a peace camp in the Rhondda Valley, Wales, many of whose participants travelled from there to inaugurate Greenham], and it all went away. I never had one of them again. (Roseneil, 2000, p. 60)

Political activism is presented as a practical means of combating feelings of despair and impotence, not only because it directed them towards the transformation of external reality, but because the process of sharing fears with others can help to lift their weight.

Why then would Segal not simply prescribe political activism as a cure for nuclear anxieties? While she does acknowledge the value of acting to change society, makes plain her concerns about psychoanalysts who failed to explicitly oppose Nazism and other forms of political repression, and underlines the importance of speaking publicly on the issue of nuclear weapons, Segal is also keen to underline that destructiveness is not only located in the external world, but also operates within every individual. As she remarked to Rose, whose 1989 essay ‘Where Does the Misery Come From? Psychoanalysis, Feminism, and the Event’ precisely articulates the problem of pitting the external against the internal, ‘[t]here is a constant interplay between the inner and the outer’ (Rose, 1990, p. 213). The fear of annihilation represented by nuclear weapons is dangerous for Segal because it can promote denial and encourage a lack of compassion. She is keen to underline that helplessness is the flipside of omnipotence, and to emphasize the danger of locating the capacity for violence only in governments and political leaders. Projecting the capability of violence onto nation-states while ignoring one’s own destructive tendencies can, she identifies, lead people to divest themselves of responsibility. Though I have expressed frustrations in this article about the disconnection between the clinical and political in Segal’s work, her insistence on the social implications of individuals’ destructive tendencies succeeds in articulating how apathy can emerge on a mass scale. As she wrote in the early 1990s:

it is not pathological to hope for a better future – for instance, for peace – and to strive for it, while recognizing how hard it is to attain, and that the opposition to it comes not only from others but also has its roots in ourselves. (Segal, 1997b35, p. 137)

For women active in the peace movement, the notion that opposition to peace could come from themselves as much as from governments might have seemed like a bizarre and politically dubious claim. But Segal was not drawing an equivalence between, say, Margaret Thatcher, and an ordinary British citizen disturbed by dreams of radioactive landscapes. Instead, she was attempting to show how fears of annihilation could precipitate denial and facilitate inaction. Her insight complements rather than contradicts the goals of peace activists, but suggests that political activism should be combined with processes of introspection; as Fraser perceived, a reckoning with the social needed to be combined with a simultaneous reckoning with the psychic.

## Conclusion

In her late essay ‘From Hiroshima to the Gulf War and After: Socio-political Expressions of Ambivalence’, written in the 1990s after the end of the Cold War, Segal describes *perestroika* as a time of hope. The Soviet Union’s status as an enemy also began to crumble in the West, a geopolitical shift that she claims to have been destabilizing to individuals, leading to forms of introspection and self-criticism brought on by collective guilt: why were all these resources spent on a now non-existent danger instead of being invested in social infrastructure? But just as the fear of annihilation during the Cold War prompted denial, so the spectacle of *perestroika* and the subsequent collapse of state socialism across the Eastern bloc prompted triumphalism: ‘rather than face guilt we turned to manic defences’ (Segal, 1997[1995], p. 136).

In a review for the *London Review of Books* of Julie McDowall’s *Attack Warning Red! How Britain Prepared for Nuclear War* (2023), Florence Sutcliffe-Braithwaite concludes by criticizing the book’s author for failing to draw parallels between fears of nuclear war in Britain during the Cold War and attitudes to climate change today. When I first read ‘Silence is the Real Crime’ I was struck by how closely Segal’s descriptions of helpless anxiety about the future seemed to match those expressed by climate activists in the present. Greenham activists’ descriptions of the hostile and physically aggressive responses of passers-by to a ‘die-in’ they participated in at the London Stock Exchange is strikingly reminiscent of contemporary responses to Just Stop Oil protestors (Cook & Kirk, 1983, pp. 40–3). The kind of resistance to knowledge identified by Segal emerges in such confrontations, where a small number of non-violent activists are treated as somehow more disruptive, aggressive and unreasonable than phenomena threatening the continued existence of life as such. The apocalyptic qualities of climate change – the scale of the threat and the concomitant scale of resistance it demands – can provoke the kind of helplessness described by Segal, and similarly require that we do not turn ‘a blind eye to reality’ (Segal, 1997[1987], p. 127).

But this is also a time of war. Russia’s full-scale invasion of Ukraine and Azerbaijan’s ethic cleansing of Armenians from Nagorno-Karabakh starkly demonstrate that the triumphalism Segal identified as accompanying the collapse of the USSR was misplaced. History did not end. Writing in 2003, Segal reflected that the end of the Cold War could have inaugurated a process of reckoning in the West, leading to an acknowledgement of domestic social injustices and ‘guilt about unnecessary wars’, particularly in Vietnam. She compares this process to a paranoid patient who begins to ‘face reality’ by giving up their former delusions. No such process occurred, however. The Gulf War, the war in Afghanistan and the Iraq War, she claims, arose partly because of this failure to collectively reckon with the bloodthirsty Cold War past:

I have grounds for believing that the deepest causes [of the Gulf War] had unconscious psychological roots. I think that war was the inevitable result of the mental constellation established after perestroika. We needed an enemy to project evil onto […] (Segal, 2003, p. 262)

Segal repeats many of her arguments about the threat of nuclear war in the context of a war being waged in the present:

The dread of annihilation, which is part of all of us, is the dread of our own death drives with their aim of disrupting, dispersing and annihilating life. Our primary defence is to project it outside and to kill it there. But it doesn’t work. (p. 264)

The solution she proposes is to refuse to ‘collude with the psychotic and disruptive functioning of our group’, which, in the context of the Iraq War, meant refusing to go along with the pro-war position taken by the British state. As I write another twenty years later, in early November 2023, Israel is relentlessly bombing Palestinian civilians and the British government and the major opposition party are refusing to call for a ceasefire in Gaza. Segal’s insistence of the importance of peace activism is worth recalling: ‘We are not like a helpless baby left alone. There is a massive protest movement throughout the world. We have strength in fraternity’ (Segal, 2003, p. 265).

The title of Segal’s ‘Silence is the Real Crime’ comes from the memoirs of Nadezhda Mandelstam, which had recently been translated into English when she first delivered her paper. They describe the profound psychological impact of living through the repressions and violence of Stalinism. Mandelstam’s husband, the poet Osip Mandelstam, was murdered in the purges in 1938. Mandelstam recalls the feelings of fearful helplessness she experienced, but concludes that even in situations in which resistance feels utterly futile, screaming is always preferable to silence:

This pitiful sound, which sometimes, goodness knows how, reaches into the remotest prison cell, is a concentrated expression of the last vestige of human dignity. It is man’s way of leaving a trace, of telling people how he lived and died. By his screams he asserts his right to live, sends a message to the outside world demanding help and calling for resistance. If nothing else is left, one must scream. Silence is the real crime against humanity. (Mandelstam, 1983, pp. 42–3)

Segal perceived the necessity of screaming against injustice even in situations in which it seemed as if the cries were not being heard.

## Footnotes

1 Thank you to Katja Diefenbach and Till Hahn for the invitation to present at the conference ‘The Politics of the Death Drive’ at the European University Viadrina, Frankfurt (Oder) in June 2022. This article was developed from the paper I gave at that conference. Thank you to Matt ffytche for providing feedback on an early draft, to Natalia Cecire, Seb Franklin, Sam Solomon and everyone who attended the work-in-progress discussions they organized in Brighton in July 2023, and, finally, to the two anonymous reviewers for their generous and insightful comments. This work was supported by the Wellcome Trust, grant number 224752/Z/21/Z.

2 On the varied and wide-ranging meanings of the term ‘British nuclear culture’, including a consideration of the shifts in attitudes and representations over time, see Hogg & Laucht (2012, pp. 479–93).

3 The manor house in which Fraser grew up is Burghfield, Berkshire, which is located just over six miles from Aldermaston, where the Atomic Weapons Research Establishment was based. Aldermaston was the site of demonstrations against nuclear armament from the 1950s onwards. CND’s first annual march to Aldermaston was in 1959. In April 1983, protestors linked arms between Greenham Common and Aldermaston to form a fourteen-mile human chain. Presumably this is the action to which Fraser is referring. See Mayer (2017).

4 I have looked through every issue of *Spare Rib* from 1979, 1980 and 1981 and couldn’t find anything that matched this description.

5 See Rycroft (1972, p. 153): ‘The capacity to distinguish between mental images and external precepts, between phantasy and external reality, to correct subjective impressions by reference to external facts. According to classical theory, the infant lacks any capacity for reality-testing. Delusions and hallucinations occurring in psychosis are defined as failures in reality-testing.’
